# Brain mapping across 16 autism mouse models reveals a spectrum of functional connectivity subtypes

**DOI:** 10.1038/s41380-021-01245-4

**Published:** 2021-08-11

**Authors:** V. Zerbi, M. Pagani, M. Markicevic, M. Matteoli, D. Pozzi, M. Fagiolini, Y. Bozzi, A. Galbusera, M. L. Scattoni, G. Provenzano, A. Banerjee, F. Helmchen, M. A. Basson, J. Ellegood, J. P. Lerch, M. Rudin, A. Gozzi, N. Wenderoth

**Affiliations:** 1 Neural Control of Movement Lab, ETH Zurich, Zurich, Switzerland; 2 Functional Neuroimaging Lab, Istituto Italiano di Tecnologia, Center for Neuroscience and Cognitive Systems, Rovereto, Italy; 3 Laboratory of Pharmacology and Brain Pathology, Neurocenter, Humanitas Clinical and Research Center - IRCCS, Rozzano, Mi Italy; 4 CNR Institute of Neuroscience, Milano, Italy; 5 Department of Biomedical Sciences, Humanitas University, Pieve Emanuele Milan, Italy; 6 F.M. Kirby Neurobiology Department, Boston Children’s Hospital, Harvard Medical School, Boston, MA USA; 7 Center for Mind/Brain Sciences (CIMeC), University of Trento, Rovereto, Italy; 8 Research Coordination and Support Service, Istituto Superiore di Sanità, Rome, Italy; 9 Department of Cellular, Computational and Integrative Biology. (CIBIO), University of Trento, Trento, Italy; 10 Brain Research Institute, University of Zurich, Zurich, Switzerland; 11 Biosciences Institute, Newcastle University, Newcastle upon Tyne, UK; 12 Centre for Craniofacial and Regenerative Biology, King’s College London, London, UK; 13 MRC Centre for Neurodevelopmental Disorders, King’s College, London, London, UK; 14 Mouse Imaging Ctr., Hosp. For Sick Children, Toronto, ON Canada; 15 Department of Medical Biophysics, University of Toronto, Toronto, ON Canada; 16 Wellcome Centre for Integrative Neuroimaging, FMRIB, Nuffield Department of Clinical Neurosciences, University of Oxford, Oxford, UK; 17 Institute for Biomedical Engineering, University and ETH Zurich, Zurich, Switzerland

**Keywords:** Autism spectrum disorders, Neuroscience

## Abstract

Autism Spectrum Disorder (ASD) is characterized by substantial, yet highly heterogeneous abnormalities in functional brain connectivity. However, the origin and significance of this phenomenon remain unclear. To unravel ASD connectopathy and relate it to underlying etiological heterogeneity, we carried out a bi-center cross-etiological investigation of fMRI-based connectivity in the mouse, in which specific ASD-relevant mutations can be isolated and modeled minimizing environmental contributions. By performing brain-wide connectivity mapping across 16 mouse mutants, we show that different ASD-associated etiologies cause a broad spectrum of connectional abnormalities in which diverse, often diverging, connectivity signatures are recognizable. Despite this heterogeneity, the identified connectivity alterations could be classified into four subtypes characterized by discrete signatures of network dysfunction. Our findings show that etiological variability is a key determinant of connectivity heterogeneity in ASD, hence reconciling conflicting findings in clinical populations. The identification of etiologically-relevant connectivity subtypes could improve diagnostic label accuracy in the non-syndromic ASD population and paves the way for personalized treatment approaches.

## Introduction

Autism spectrum disorder (ASD) is a neurodevelopmental condition marked by social, communication and behavioral challenges often accompanied by additional co-morbidities that together negatively impact the quality of life of affected individuals and their families. The high heterogeneity of clinical presentation and underlying pathophysiology pose a substantial challenge for early diagnosis and effective treatments [1, 2]. Among the multiple and diverse etiological factors associated with ASD [3], genetic alterations appear to be by far the largest contributors to ASD risk [4]. Several studies have revealed that genetic variants associated with ASD cause cellular alterations linked to abnormal neuronal circuit wiring and function, leading to aberrant developmental trajectories (reviewed by [5]). Hence, circuit and network dysfunctions are thought to directly underlie onset and severity of ASD symptoms.

Within this framework, abnormalities in the coordinated functional interactions of brain networks, or brain connectivity, might therefore represent a defining hallmark of ASD. This theoretical view was first inferred from fMRI measurements of cortical activation collected during different cognitive tasks [6]. Since then, a growing number of studies in idiopathic [7, 8] as well as syndromic forms of ASD [9–12] has suggested that aberrant connectivity in ASD could be detected by resting-state fMRI (rsfMRI). However, whilst a recent aggregate analysis has revealed a putatively reproducible mosaic pattern of atypical connectivity in ASD [13], the heterogeneity in rsfMRI connectivity findings across ASD cohorts is considerable, and unlikely to reflect technical or procedural discrepancies in imaging acquisitions and analysis [2, 12, 14]. Hence, one outstanding question in the field is whether ASD can be associated with a univocal, diagnosis-specific signature of dysfunctional brain connectivity that is common to the whole spectrum, or whether clinical heterogeneity is the sum of distinct and separable signatures of network dysfunction.

rsfMRI connectivity studies in etiologically homogeneous ASD populations have started to link disease-causing genetic alterations to specific functional connectivity aberrancies, highlighting putatively distinguishable, etiology-specific connectivity changes. For example, subpopulations harboring well-characterized genetic mutations such as chromosome 16p11.2 deletion [11], neurofibromatosis type 1 mutations (Nf1) [15] or Fragile-X (Fmr1) syndrome [9, 16] are characterized by non-overlapping patterns of dysconnectivity. Similarly, mTOR-related synaptic surplus has been recently linked to a specific cortico-striatal hyper-connectivity signature [17]. These observations suggest an interpretative framework in which ASD-relevant etiological and genetic risk factors could lead to distinct network signatures of brain dysfunction, thus explaining heterogeneous connectivity alterations observed in clinical cohorts. Crucially, this model would also explain why so far, no unequivocal ASD-specific signature of abnormal connectivity has been identified.

To test this hypothesis, we introduce the Autism Mouse Connectome (AMC) study, a bi-center initiative dedicated to collecting and analyzing rsfMRI in multiple ASD-relevant mouse mutants under well-controlled and highly reproducible experimental conditions [18–20]. Our work leverages recent advances in cross-species rsfMRI imaging, revealing that rsfMRI is exquisitely sensitive to network alterations caused by ASD-related etiologies, reconstituting patterns of network dysfunction in corresponding human ASD cohorts [11, 16], [21–26], [17, 21–26]. By comparing connectivity alterations caused by 16 ASD-related genetic and etiological factors, we found that ASD etiologies cause a wide spectrum of connectivity abnormalities that can be clustered into a discrete set of prevailing subtypes. Our results underscore a pivotal contribution of etiological variability to connectivity heterogeneity in ASD and reveal a set of prominent cross-etiological network dysfunction modes of high translational relevance for ASD.

## Results

### Animal models

The AMC collection includes scans from published literature that have been retrospectively aggregated and additional unpublished work (see Table 1). The data were all acquired following strict anesthesia procedures under mechanical ventilation which are documented elsewhere [19, 27–29]. Our previous work shows that these procedures preserve heart rate and blood oxygenation within a physiological range throughout the experiment. Importantly, the employed regimens have been shown to produce rsfMRI networks that are topographically indistinguishable [30], tightly related to the underlying axonal architecture of the mouse brain [31, 32] and characterized by rich dynamics [33, 34]. In total, it contains resting-state fMRI scans of 350 mice from 16 distinct cohorts. Ten cohorts were collected at ETH Zürich (Switzerland) and six cohorts were collected at IIT Rovereto (Italy). Data from two independent cohorts of CNTNAP2 were collected at both sites.

**Table 1:** Available datasets.

ASD mouse model	Control strain	Sex	Age (weeks)	Behavioral traits*	Construct validity^†^	References	Scan site
16p11.2 (df/+)	129/C57BL6J	males	18–20	3, 5	c	[11]	IIT
BTBR T+tf/J	C57BL/6 J	males	26	1, 2, 3, 4, 5	d	[21]	IIT
CDKL5 KO	C57BL/6 J	males	23–25	3, 5	b, f	–	ETH
CDKL5 Het (+/−)	C57BL/6 J	females	17–19	3, 5	b, f	–	ETH
CHD8	C57BL/6 J	mixed	15–18	3, 4, 5	a	[26]	IIT
CNTNAP2_ETH (−/−)	C57BL/6 J	mixed	14–16	1, 4, 5	a, f	[16]	ETH
CNTNAP2_IIT (−/−)	C57BL/6 J	males	13–14	1, 4, 5	a, f	[23]	IIT
En2 (−/−)	C57BL/6 J	mixed	12–16	1, 4, 5	a, e, d	[24]	ETH
FMR1.1 (−/y)	C57BL/6 J	males	14–16	1, 2, 3, 4	b, f	[16]	ETH
FMR1.2 (−/y)	C57BL/6 J	males	13–15	1, 2, 3, 4	b, f	[22]	ETH
IL6	C57BL/6 J	mixed	14	1, 4	d	[76]	ETH
Mecp2 Het (+/−)	C57BL/6J	females	12–14	1, 4, 5	b, f	–	ETH
SGSH (−/−)	C57BL/6 N	mixed	20–22	5	b	–	ETH
SHANK3b ex4-9	C57BL/6 J	males	19–21	1, 2, 3, 4, 5	a, f	[25]	IIT
Syn2	C57BL/6 J	males	25–31	1, 2, 3, 4	f	[56]	IIT
TREM2 (−/−)	C57BL/6 J	mixed	12–15	3, 4	d	[37]	ETH

*Behavioral traits related to ASD reported by others in selected models: (1) social behavior deficits; (2) reduced vocalizations; (3) repetitive behavior; (4) cognitive behavior; (5) motor performance.

^†^Construct validity includes: (a) Genetic association; (b) Related syndrome; (c) Autism copy number variants; (d) Autistic-like behavior; (e) Serotonin-related phenotype; (f) Synapse related.

All mouse mutants fulfilled the following criteria: (1) the genetic modification resembles/relates to a genetic alteration found in individuals with ASD as listed in the SFARI gene database (https://gene.sfari.org/autdb/); (2) the mouse strain has been shown to mimic at least one of the core behavioral phenotypes of ASD; (3) mice are inbred with C57BL/6 J or /6 N mice as control strain to reduce genetic heterogeneity across models; (4) each cohort included mice with the genetic alteration and wild-type control littermates. In addition, we included: (i) a model for environmental ASD risk factor, i.e., maternal exposure to interleukin-6 (IL-6); the maternal injection of IL-6 at later stages of pregnancy (E12,5-E15) is a well-established model of Maternal Immune Activation (MIA), and the relative offspring show major behavioral defects which include alterations in sensory-motor gating, attention and sociability, all clear hallmarks of ASD; [35, 36] (ii) a model for TREM2 deficiency; TREM2KO mice are characterized by defects in microglia-dependent synaptic pruning activity which results in excessive glutamatergic synaptic connections accompanied by autistic-like behavioral phenotype [37] (iii) a model of congenital agenesis of the corpus callosum, a neuroanatomical trait associated with increased prevalence of ASD; [38] inbred BTBR mice are often employed as a rodent model for “idiopathic” ASD and exhibit profound behavioral and neurofunctional alterations of high translational relevance [39–41].

### Between-cohorts and between-sites connectome consistency in control animals

**Fig. 1:**
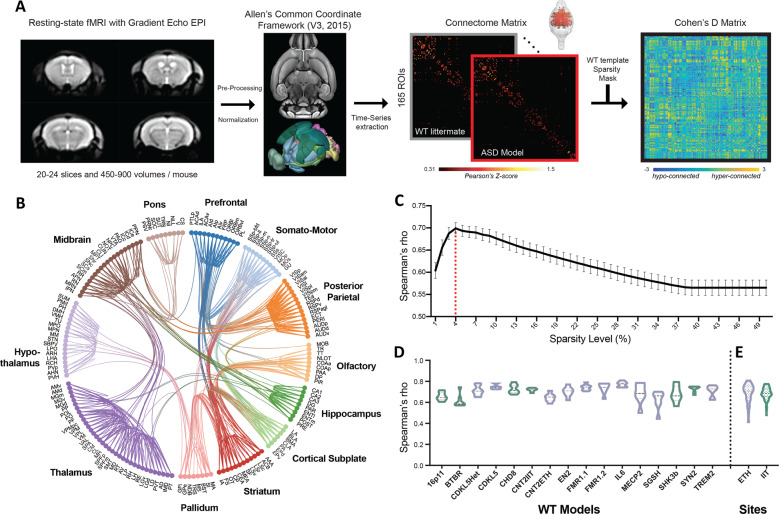
WT datasets show comparable connectome representation across recording sites. **A** Schematic of the experimental pre-processing and processing pipeline. An arbitrary sparsity thresholding is taken from the averaged template connectome and then applied to each cohort’s Cohen’s D matrix. **B** Circos plot of the mouse brain connectome from 112 rsfMRI wildtype datasets, 4% sparsity threshold, highlights a complex network of distributed short- and long-range connections (number of edges = 545). **C** Quantification of similarity across all WT mice at different sparsity levels (1–50%). Maximum averaged Spearman’s rho was found at 4% sparsity. **D** Distributions of ranked similarity in connectome representation (Spearman’s rho) between wildtype of each cohort and the group average. **E** Same data as in (**D**) but grouped within each of the two measurement sites (ETH, IIT). Both nonparametric testing and Bayesian repeated-measures ANOVAs show that datasets from WT mice exhibit comparable connectome representation independently from recording site. Wilcoxon matched-pairs signed rank test, *p* = 0.2749. Bayesian factor BF-H0 (null) = 7.57.

rsfMRI data from all cohorts were commonly preprocessed using a standard minimal-preprocessing pipeline and normalized to the Allen Mouse Brain Common Coordinate Framework (CCFv3) (Fig. 1A). Our first goal was to test the assumption that scans from wild-type (WT) control animals would show comparable functional connectome characteristics, so that data from the two sites and different cohorts can be directly compared. In order to ensure an unbiased edge selection across sites and cohorts, we selected an equal amount of data from the two sites (*n* = 112) randomly taken from the different cohorts and computed an averaged mouse functional connectome template. This template is then used to select relevant edges (i.e., sparsity mask) for subsequent analyses (Fig. 1B).

The similarity between the connectome representation of each individual WT mouse and the group average template—expressed by Spearman’s ranking coefficient (Rho)—is used to quantify consistency of the data (larger Rho refers to larger similarity between WTs). This parameter was evaluated for a range of sparsity levels taken from the functional connectome template, which varied from keeping only the top 1% of the strongest and positively correlated edges to keeping up to 50%. Maximum similarity between datasets was obtained at 4% sparsity (545 edges) (Fig. 1C). This indicates that the functional connectome in the mouse is maximally similar between all data sets when considering the 4% strongest and positively correlated edges. This level of sparsity was applied to all subsequent analyses. Similarity indexes between the individuals of each cohort and the group average of all WTs are shown in Fig. 1D. The results show a marked resemblance across animals (mean Spearman’s rho: 0.699 ± 0.06) and low variability between cohorts.

Next, we tested whether the data across the two sites exhibit significant differences, for example, due to differences in environmental conditions in which mice were raised, experimental protocols including anesthesia or data acquisition hardware. Importantly, we found no significant differences in mean similarity between data collected at the two sites (Wilcoxon matched-pairs signed rank test, *p* = 0.2749, Fig. 1E). In addition, Bayesian repeated-measures ANOVAs revealed a Bayes Factors in favor of the null hypothesis (BF-H0, i.e., data from the two sites are not different) of 7.57 versus a BF that rejects the null hypothesis of 0.13 (BF-H1, i.e., data from the two sites are different). We next used a machine learning (ML) approach to assess whether a classifier trained with half of the WT data (train-set) could distinguish the site of origin in the other half of the data (test-set). Our logic is that if the datasets of the two sites are similar, a ML classifier should not be able to distinguish the site of origin and the test predictions should be close to random predictions. The train-test process was repeated 100 times at multiple sparsity levels (2–20%). Supplementary Fig. 1 shows that a linear support vector machine (LSVM) failed to predict the site of origin of the test dataset above chance level, for all sparsity levels and across a broad range of parameters. Overall, these data clearly indicate that the connectome representation is comparable in WT animals irrespective of which site performed the rs-fMRI measurements (ETH and IIT).

### Low-dimensional representation of connectivity changes in individual animals reveals a wide spectrum of alterations

Autism is commonly defined as a “spectrum” of neurodevelopmental disorders, owing to the wide range of etiologies and pathophysiological factors that has been associated with this condition. It is, however, unclear whether abnormal functional connectivity faithfully mirrors the ASD etiological variability or converges onto a single common signature of circuit dysfunction. To disambiguate these two opposing possibilities, we implemented a dimensionality reduction algorithm and mapped individual mouse connectivity data into the same coordinate system. After determining connectivity deviations in the ASD mutants relative to their own WT controls, connectivity abnormalities of each animal were projected onto a low-dimensional space using an unsupervised manifold learning technique (Uniform Manifold Approximation and Projection, UMAP), which preserves the global data structure and the local neighbor relations better than other existing methods such as Principal Components or t-distributed Stochastic Neighbor Embedding (t-SNE) [42]. Point-to-point distances in UMAP plots were then used to interpret the continuity of the data subsets and identify similarities in “connectopathy” between individual animals.

**Fig. 2:**
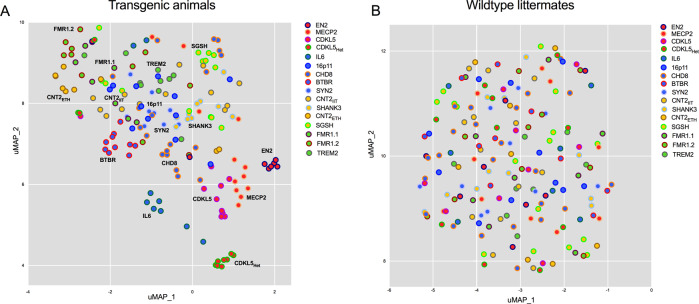
ASD-related etiologies define a continuous connectivity landscape. **A** Uniform manifold approximation and projection (UMAP) 2-dimensional embedding of the connectome data from 176 individual ASD-related model animals of the AMC dataset. Individual data are *Z* scored and normalized to the average cohort’s WT control population. **B** UMAP embedding using data from 174 wildtype animals did not lead to clear clusters across both groups and site/anesthesia. The color of the elements represents the model’s cohort. uMAP parameters: n_neighbours = 10 min_dist = 0.1.

Notably, the resulting map revealed a continuous spectrum of connectivity abnormalities across which individual etiological clusters could be located and identified (2D representation, Fig. 2A). This is in stark contrast to the UMAP representation of wild type animals, in which no cluster between cohorts of animals is visible (Fig. 2B). The analysis of the Euclidian distance between points in the low-dimensional embedded space (corresponding to the (dys)connectivity profiles of individual animals) revealed that distances were shorter between individuals of the *same* ASD cohort (reflecting more similar connectivity alterations) than between individuals of different ASD cohorts (for 2D embedding, Euclidean distance within-groups = 1.5 ± 0.56; between groups = 2.49 ± 0.45, *T* test *p* = 2.55e^−8^, Supplementary Fig. 2). This feature was preserved when using a higher number of dimensions in the embedding space (three to ten dimensions) and was not present in the analysis of Euclidean distances in wildtype animals (Supplementary Fig. 2). Importantly, the overall distribution of subjects across this low-dimensional space defined a cross-etiological continuum within which some ASD etiologies appeared to be clustered and distinguishable (e.g., FMR1, IL-6, CDKL5, BTBR) while others were widespread and partly overlapping (CHD8, CNT2, 16p11). Taken together, these results suggest that (i) the underlying etiology determines the similarity in connectopathy between individual animals and their position in the embedded space; (ii) the resulting distribution of connectivity profiles defines a continuous landscape of connectivity alterations, arguing against the idea of a common pattern of connectivity abnormalities across all the probed models. Importantly, dimensionality-reduction method such as UMAP supports an inverse transform that can approximate how a new sample—or new animal model—would connect to a specific position in the embedding space. This feature is particularly interesting for future research that aims to compare new genetic models within the two-dimensional reference framework of this first AMC study.

### ASD-specific connectivity signatures can be segregated into cross-etiological clusters

**Fig. 3:**
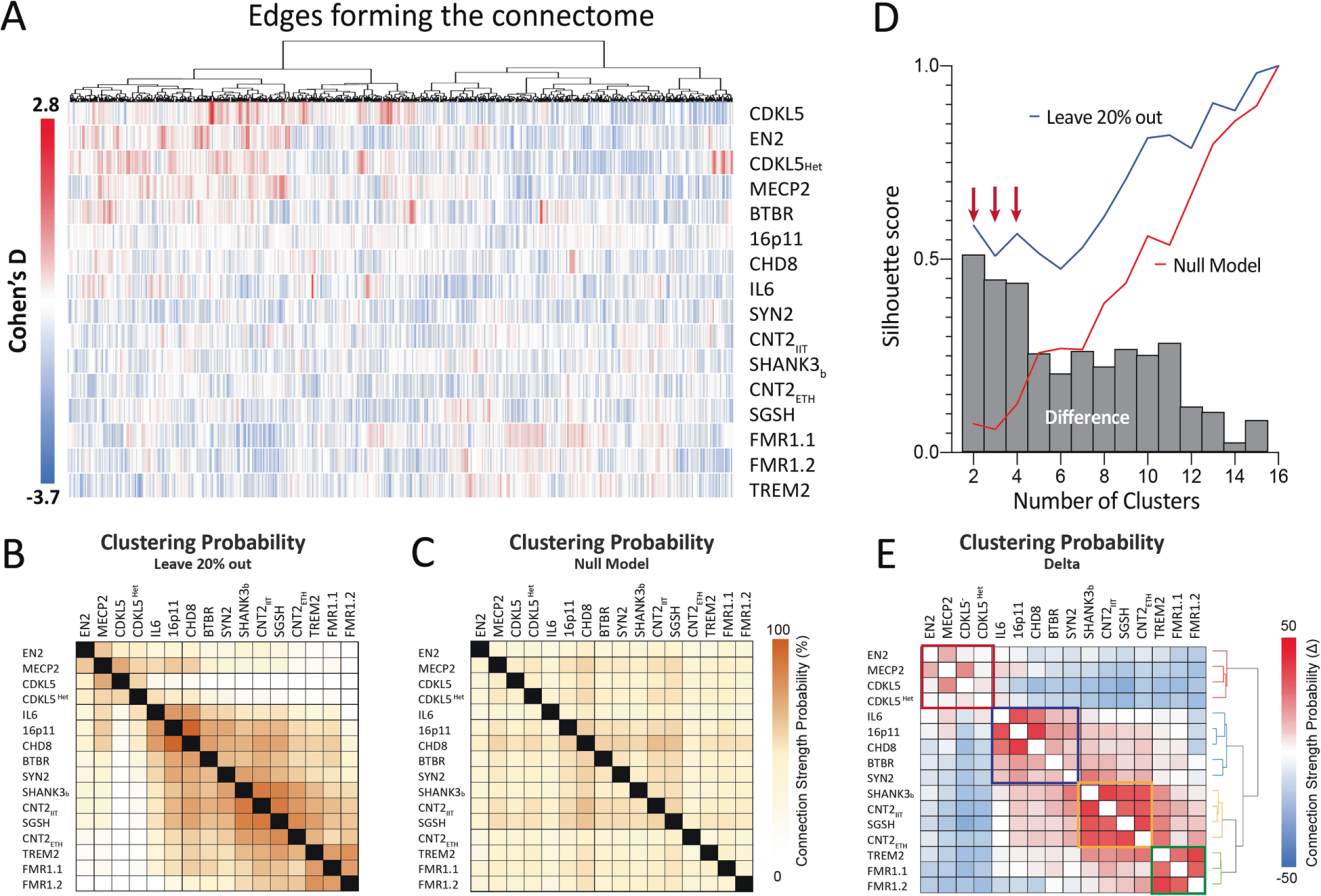
Functional connectivity signatures can be grouped into four cross-etiological clusters. **A** Functional connectivity aberrances in the 16 ASD mouse cohorts. The heatmap displays the effect size (Cohen’s D) differences in connectivity strength between the 16 different mouse models and their specific WT controls for each of the 545 different edges across the connectome. Red represents over-connectivity compared to control and blue represents under-connectivity. The dendrogram on the *x* axis represents the correlation between edges. **B** Gaussian Mixture Model revealed similarities across mouse cohorts. Clustering probability (%) is measured based on the proportion of time that two cohorts belong within the same cluster over the 1000 bootstrapped samples using the leave-20%-out criteria. **C** Clustering probability of the null model generated by randomly assigning knockout and wildtype labels in each cohort 1000 times. **D** Silhouette score measured mean intra-cluster distance and the mean nearest-cluster distance in both the real and null distributions for different cluster solutions (*n* = 2, 3, … 16). High silhouette score differences are found in the 2, 3 and 4 cluster solutions. **E** The hierarchical clustering using 4-cluster solution segregated the models into specific groups depending on their connectivity similarity.

In the previous analysis, we have shown that different autism-associated genetic etiologies define a pseudo-continuous landscape of connectivity alterations. However, one outstanding question is how well a “categorical” diagnostic approach, for example based on quantitative and robust clustering methods, can effectively model this complexity and define common connectivity signatures across mutants [43]. To answer this, we first assessed the deviations in connectivity strength for each of the edges between the mutant mice and their control littermates by their effect size, i.e., Cohen’s D. The Cohen’s D was then entered into a matrix (545 edges × 16 cohorts) representing connectivity alterations for each of the 16 models (Fig. 3A). In keeping with the results of our low dimensional mapping, a visual inspection of the obtained matrix revealed the presence of different connectivity profiles, entailing spatially distributed rather than focal patterns of abnormal connectivity. Some of the abnormal patterns exhibited opposing features, such as over- and under-connectivity within the same pair of nodes (see top left and bottom left section of matrix in Fig. 3A). The resulting matrix was fed into an unsupervised Gaussian Mixture Model (GMM) clustering algorithm to determine the general structure of the data and to establish putative similarities between the different mouse cohorts. In order to verify the reproducibility and stability of these clusters, we measured the proportion of time that two cohorts were clustered together using a bootstrap procedure (Fig. 3B). Therefore, we randomly selected 80% of the data within each cohort, calculated the Cohen’s D for all edges, and applied the clustering algorithm (1000 times). We also determined a null distribution by repeating this procedure on data that were randomly shuffled between mutants and WT (also 1000 times, Fig. 3C). To determine the optimal number of clusters, we computed the silhouette score for a number of possible cluster solutions (ranging from 2 to 16) using GMM. The silhouette score considers the distance between one and all other cohorts within the same cluster (cohesion), and the distance between one and all other cohorts in the next nearest cluster (separation). High silhouette values reflect high cohesion and high separation indicate that cohorts are well matched to their own cluster and poorly matched to neighboring clusters. Since the GMM procedure is not deterministic, we ran 1000 fits for each number of clusters. The difference in silhouette scores between the bootstrapped and the null model was used to determine the best cluster solution(s). The bar plot in Fig. 3D shows a high difference score for two, three and four-cluster solutions. In order to preserve most of the complexity in the data, we kept the four-cluster solution for the following analyses. Next, we calculated the connection strength probability between each pair of cohorts (i.e., how often they have been assigned to the same cluster during bootstrapping; Fig. 3B, E). When grouping the cohorts into four clusters, the cluster with the strongest within-cohort connections is formed by TREM2 and both Fmr1 cohorts, which were grouped together on average 74% of the time. This was followed by a cluster that includes CNTNAP2, Shank3b, and SGSH (on average grouped 73% of the time). The two CNTNAP2 models scanned at different institutes were clustered together 75% of the time. The other two clusters include (i) CDKL5 KO, CDKL5 Het, Mecp2 Het and EN2 (59%, with CDKL5 KO and CDKL5 Het being clustered together 67% of the time) and (ii) BTBR, IL6, 16p11 and CHD8 (on average grouped 51% of the time). The hierarchical representation of the clustering connections between cohorts is shown in Fig. 3E. UMAP representation of individual animals color-coded by their respective cluster is shown in Supplementary Fig. 3. Taken together, these findings suggest that ASD-specific connectivity signatures can be segregated into distinguishable cross-etiological clusters.

### Cross-etiological cluster stability

In order to evaluate the stability of the identified clusters, we reanalyzed the data with a wide range of experimental parameters. First, we computed the clustering probabilities across the models at multiple sparsity levels (2%, 3%, 5%, 6%, 10% and 20%). For each level, we calculated the probabilities for each pair of models to be clustered together using our GMM approach repeated 1000 times. The clustering probabilities were compared against an appropriate null model of data with the same sparsity level but with randomly shuffled labels. Overall, we found strong consistency between cluster probabilities obtained using a 4% sparsity threshold and the other levels (Pearson’s rho ranged between 0.962 and 0.878, Supplementary Fig. 4). These results suggest that our clustering probabilities are reliable and robust against this parameter.

We then verified whether different ROI sizes could have influenced our results, as larger regions can lead to stronger connectivity, such that connectivity between smaller regions could be disproportionately affected. Therefore, we conducted a new analysis using 165 ROIs with equal size (i.e., a sphere of 3 voxels in diameter) and centered on the coordinates of the center of gravity of each region of the Allen Brain atlas. The results of this re-analysis led to a very similar clustering probability between the two parcellation schemes (Pearson’s *r* of 0.9620, *p* < 0000, Supplementary Fig. 5).

Finally, we re-evaluated the presence of clusters between models considering ROIs from both cerebral hemispheres. In previous analyses, we focused on intrahemispheric connectivity and averaged BOLD signals between homologous ROIs of both hemispheres to increase the signal-to-noise ratio. Our rationale for this choice is that there is a strong symmetry between the connectivity profiles in the two hemispheres in wildtype mice (as reported in a recent meta-analysis by Grandjean et al., 2020). However, it is possible that some transgenic models suffer from interhemispheric connectivity deficits, which could lead to an asymmetry in connectivity abnormalities. To check if this is relevant, we performed an additional analysis using a new parcellation scheme of 165 ROIs taken separately in each hemisphere (i.e., 330 ROIs in total). First, connectome analysis in wild type mice confirmed that the similarity between the data sets is maximal for a sparsity of 4% (Supplementary Fig. 6A). Next, we applied our clustering approach and found a strong consistency with the previously described clustering results, with the only difference evident in the BTBR model. Namely, inclusion of inter-hemispheric edges revealed that BTBR mice are less likely to be grouped with any other mouse model, including IL6, CHD8, and 16p11 as we previously reported (Supplementary Fig. 6B–F). This is likely due to the fact that the new parcellation scheme includes cortico-cortical interhemispheric connections, which are known to be severely compromised in acallosal BTBR mice.

### Common connectome deviations across etiologies define distinguishable ASD connectivity subtypes

**Fig. 4:**
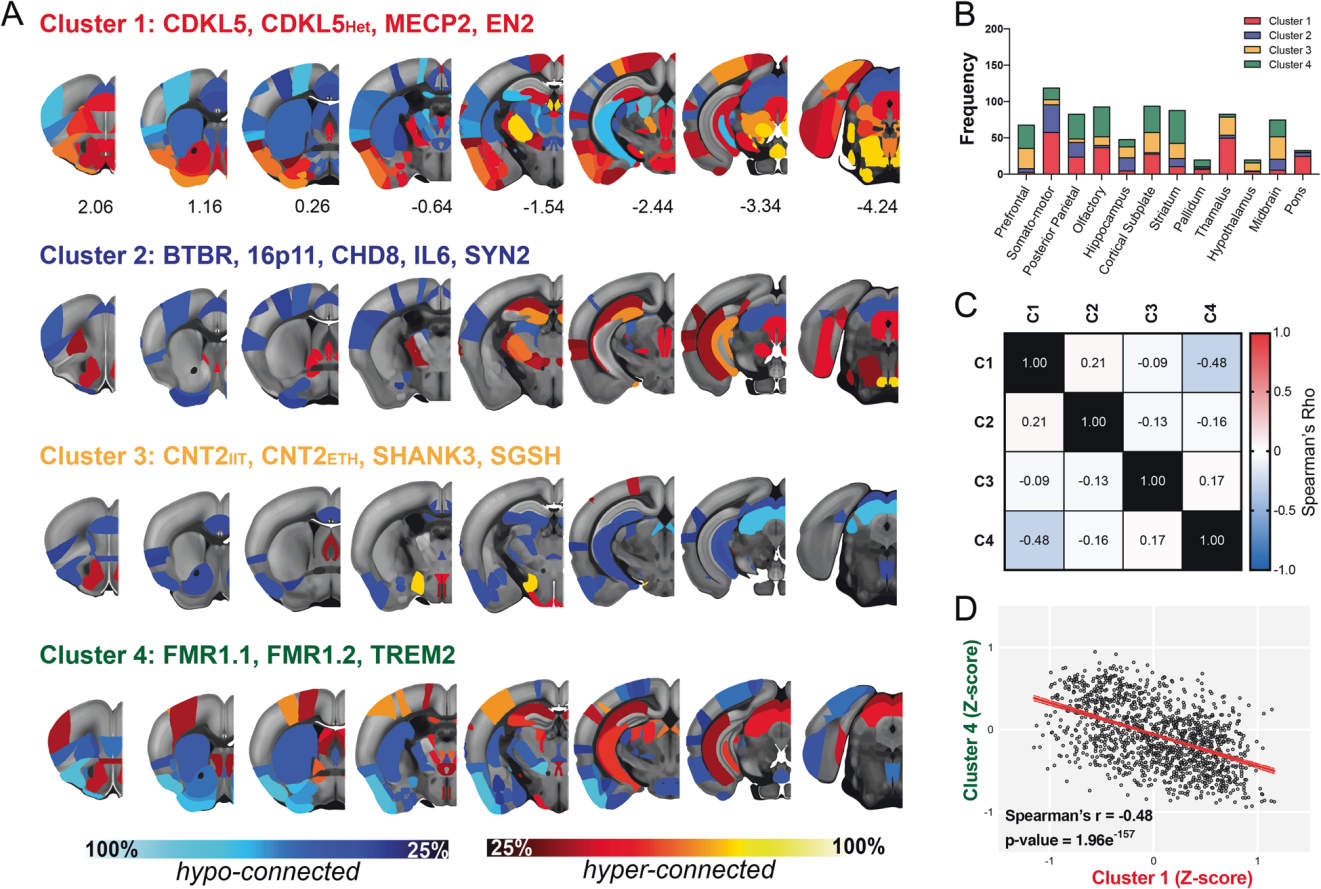
Anatomical representation of connectivity deficits in the four subtypes. **A** Rendering of regional connectivity deficits in the four clusters at the node level, revealing a heterogeneous set of brain areas with prominent over- and under-connectivity. Data are visualized in Allen Mouse reference space. **B** Number of connections (displayed as stacked frequencies) that exhibited abnormalities at the parent level. **C** Correlation matrix between all clusters, considering all 545 edges. **D** A significant negative correlation was found between Cluster 1 and Cluster 4. Spearman’s rho = −0.48, *p* = 1.96e^−157^.

Our results so far indicate that mutations in different ASD-related genes are associated with distinctive connectivity aberrances. Some of these ASD mouse models, however, exhibited similar profiles of abnormal connectivity that can be grouped and categorized into a small number of cross-etiological subtypes. To further pinpoint the spatial location of these connectivity alterations within each subtype, we used a general linear model (GLM) with nonparametric permutation testing to evaluate for connectivity abnormalities when all mice within one cluster were pooled. The results are shown in their anatomic structure (node-level comparison, Fig. 4A). The original structure (edge-edge comparison) and their macro-area (parent-level comparison) are shown in Supplementary Fig. 7.

As expected, the distribution of significant over- and under-connected edges (*p* < 0.05) varied across the four clusters, defining four distinct connectivity signatures (Fig. 4A). The first cluster was characterized by under-connectivity in insula, somato-motor cortices, anterior cingulate, caudoputamen, hippocampus, colliculus and increased connectivity between areas in prefrontal, orbitofrontal, piriform, visual cortices, amygdala, ventral posterolateral thalamus and pontine nuclei. The second cluster showed under-connectivity between cortico-striatal areas and inferior colliculus but increased connectivity between ventral orbital, lateral septal nuclei cortex and hippocampus. The third cluster displayed under-connectivity of anterior cingulate, insula, hippocampus and thalamic VPM and only a moderate over-connectivity in the accumbens shell and hypothalamus. The fourth cluster exhibited under-connectivity between piriform and olfactory-related areas, striatum and thalamus (polymodal-associated areas) and over-connectivity in hippocampus and hypothalamus.

Across all clusters, the regions showing higher vulnerability to abnormal connectivity (independent of directionality, i.e., over- or under-connectivity) were the somatomotor regions, olfactory and cortical subplate. Pallidum, hypothalamus and pons were the least affected areas (Fig. 4B). Interestingly, some of the observed connectivity features across clusters appeared to exhibit opposing configurations. Such effect was apparent in Clusters 1 and 4, where diverging connectivity alterations in cortical and hippocampal areas were observed. This notion was quantitatively corroborated by formal testing of the independence of the clusters, in which we compared the connectivity profiles in all four clusters using ranked statistics (Fig. 4C). This analysis revealed no correlation among the cluster, with the predicted exception of a strong negative correlation between edges in clusters 1 and 4 (Spearman’s rho = −0.48, *p* = 1.96e^−157^), i.e., edges that were over-connected in cluster 4 were under-connected in cluster 1 (and vice versa). This data suggests that these etiologies affect the same networks, but connectivity deviates in opposite directions (Fig. 4D).

The previous analysis described which anatomical networks exhibited connectivity abnormalities in either of the four clusters. Next, we sought to identify whether we could isolate aberrant connectivity patterns that are unique for a given cluster, hence defining which anatomical connections are preferentially affected in each subtype. To this end, we grouped the animals belonging to each cluster and compared them with the animals belonging to the other clusters using non-parametric permutation tests. In line with the results of our regional mapping, this analysis confirmed the presence of functional connectivity alterations that are unique to each cluster (Supplementary Fig. 8).

Finally, we probed the presence of a connectivity signature that could emerge at the population level, across all the etiologies tested in our study. This was done by testing all genetic models against all wild-type littermates. Consistent with the large heterogeneity in the (dys)connectivity signatures mapped, this analysis revealed only a small number of connections (*n* = 24, 4.4% of the selected edges) that survived correction for multiple comparisons (Supplementary Fig. 9). Under-connectivity was observed between somatomotor areas, anterior insula, caudoputamen, fundus of striatum, endopiriform nucleus and claustrum, including a set of substrates that is not representative of the wide area of substrates identified in our cluster analysis. These results seem to indicate that there are only very few connectivity deficits that are common among the models, arguing against the presence of a common brain signature of dysfunction that is characteristic of the autism spectrum.

## Discussion

The absence of reliable and specific cross-etiological molecular or genetic biomarkers for ASD [1, 44] have prompted research into the use of functional neuroimaging readouts as possible point of convergence for a diagnostic or prognostic characterization of ASD. However, until now connectivity studies in ASD patients have revealed highly heterogeneous and inconsistent results, which has fueled discussion regarding the clinical utility of imaging markers in ASD [45]. Here, we capitalized on recent advances in rodent rsfMRI [19, 20, 28, 46–48] to measure connectivity across 16 different ASD mouse models. We aimed to coarsely approximate the heterogeneity and etiological complexity of ASD, with the advantage of having a tight control of environmental factors, a priori information on the nature of these etiologies, and a well-matched reference population for connectivity mapping (i.e., WT littermate mice), hence controlling for major confounding factors in clinical neuroimaging research.

Our approach resulted in three important findings that bear high translational relevance for ASD research. First, we found that all models are characterized by significant alterations in brain functional connectivity entailing multiple, spatially distributed patterns of abnormal connectivity, rather than focal atypicalities. This finding is consistent with human literature [2], and suggests that altered large-scale inter-areal communication is a hallmark endophenotype in ASD across etiologies, substantiating prior conceptualizations of ASD as a brain “connectopathy” [49, 50]. Second, our data exhibited a broad spectrum of connectivity aberrancies even when mapped onto a low-dimensional landscape, indicating the absence of a prominent consensus pattern of abnormal connectivity across etiologies. This observation is of great importance in light of the ongoing debate as to the origin and significance of connectivity changes described in rs-fMRI of ASD individuals. Together with the results of analogous cross-etiological clustering of brain structure in rodent ASD models [51], our data argue against the existence of a reliable and specific autism-specific brain signature, and suggest that the substantial heterogeneity underling the neurobiology is a plausible key driver for the multiple inconsistent connectivity findings in clinical ASD. Finally, a third central conclusion of our analysis is that different ASD etiologies can be grouped into a small number of “families” or “clusters” which exhibit common atypicality in specific brain connections, defining a putative set of network dysfunction ASD subtypes [2]. Importantly, these patterns (Fig. 4, Supplementary Fig. 7) are not identifiable when analyzed across all mouse models (Supplementary Fig. 9), corroborating the notion that the ASD connectivity landscape is composed of a set of etiology-specific connectivity aberrances converging onto a small set of common networks dysfunction modes. These findings strongly support current efforts of using neuroimaging per se [2] or as part of multidimensional decompositions [52], to deconstruct ASD heterogeneity into homogenous subtypes, and suggests that rsfMRI is sufficiently sensitive to ASD-related pathology to serve as a reliable categorization axis in these efforts.

Interestingly, we found that connectivity aberrations in two of these clusters affect similar networks, but are opposite in direction, i.e., networks that are over-connected in one cluster are under-connected in the other. As discussed above, this finding may be critical for explaining inconsistencies in human studies that consider all ASD subjects as a single group, without considering the underlying etiological variability. It is indeed conceivable that the combined effect of these two opposite subtypes may reduce—if not cancel out entirely—the ability of rsfMRI to identify significant group-level changes. The exact clinical significance and occurrence of these subtypes remain to be established. However, it is tempting to speculate that such a scenario may partly account for previous reports hinting at the lack of robust connectivity alterations in ASD cohorts [12, 53].

Prior structural and functional investigations in some of the models we imaged here suggest a non-dyadic relationship between morphoanatomical changes and rsfMRI connectivity, preventing a direct comparison between our findings, and those obtained using morphoanatomical imaging by Ellegood and colleagues [51]. For example, 16p11.2 del mice exhibit basal forebrain and hippocampal gray matter volume reductions [54, 55], but preserved connectivity in these areas, and altered functional coupling in fronto-cortical regions [11]. Similarly, functional connectivity alterations in SynII knockouts are not associated with any discernible morphoanatomical volumetric changes [56], and acallosal BTBR mice exhibit widespread reduction in cortical thickness but largely preserved cortico-cortical connectivity [21, 57]. These results argue against a direct contribution of structural neuroimaging alterations to our connectivity findings, and suggest that functional and morphometric readouts represent two distinct and complementary dimensions for autism sub-typing.

While the relatively low number of mutations examined here does not permit speculation about the significance of the etiological groups identified in terms of molecular pathways, transductional cascade and common behavioral deficits, it is encouraging to note that some associations may be mechanistically meaningful. These include, for example, the observation that both homozygous and heterozygous CDLK5 mutants were grouped together with MECP2, consistent with the hypothesis that these models share the same molecular dysfunction since CDKL5 is a kinase able to mediate MeCP2 phosphorylation [58, 59]. However, for the most part, the group of mutations clustered together are highly heterogeneous and do not appear to represent any obvious signaling or molecular cascade. This finding is, however, not surprising, as temporal pleiotropy and developmental timing are essential factors which might cause similar patterns of network dysfunction even if the affected molecular mechanisms are seemingly distinct [5]. Notably, our clustering approach did not distinguish between mutants with a clear genetic association with ASD versus mutants that code for syndromes which are only partially related to autism. This indicates that the mapping between distinct ASD etiologies and the brain connectivity subtype is complex and most likely driven by a multitude of different biological, environmental and developmental factors. Future expansions of our dataset to a sufficiently large number of genetic etiologies which are more representative of the complex genetic landscape of ASD [60] may be envisaged to further explore whether alterations in known molecular pathways converge on similar network alterations. The expansion of the AMC database might not be limited to ASD mutants but could also include mouse models of other neurodevelopmental disorders to identify whether similar alterations of brain connectivity manifest across diagnostic categories. Similarly, the addition of multidimensional phenotyping data to include behavioral readouts may help to assess the translational significance of the mapped brain (dys)connectivity signatures, as it might reveal their relationship with core deficits that characterize ASD. It should, however, be noted that the relationship between connectivity findings and ASD-behavioral traits remains contentious: while prior mouse work has revealed a possible link between network-specific dysconnectivity and specific ASD-related behavioral traits, recent clinical meta-analyses suggest that rsfMRI connectivity is a poor predictor of ASD symptomatology [13]. Our observation of diverging connectivity across models characterized by partly-concordant behavioral deficits is consistent with the notion that ASD-related behavioral dysfunction cannot be monotonically represented in connectional terms. This aspect, however, does not diminish the mechanistic relevance of our connectivity endophenotypes for functional sub-typing and cross-etiological clustering. Further investigations are required to clarify the significance of our connectivity fingerprints and their relationship with ASD-related behavioral dysfunction.

In this respect, it is interesting to note that somato-motor, insular and striatal networks appeared to be among the most frequently affected substrates across mutations and clusters. This finding is not surprising in light of the sensory perception and motor-related impairments commonly described in many of the examined models. For example, both Engrailed 2 and Fmr1 knockout mice are characterized by blunt encoding of tactile stimulation frequency, increased sensitivity to somatosensory stimuli and larger size of receptive fields in the somatosensory cortex [24, 61, 62]. Similarly, hyperactivity and excessive self-grooming have been described in many models included in this study, most notably Shank3 [63] and CNTNAP2 [64], and repetitive/restricted behavior has also been observed in En2, Fmr1, BTBR and MECP2 mutants [65], recapitulating hallmark ASD symptoms commonly related to compromised fronto-striatal-motor signaling [66–68]. Future research employing targeted cellular and circuit manipulations in rodents combined with similar fMRI recordings [69, 70] may crucially uncover the bases of these network-level alterations, their behavioral significance, and their translational relevance with respect to analogous measurement in clinical populations [11, 71].

In conclusion, our cross-etiological analyses of functional connectivity in 16 ASD models revealed a broad spectrum of functional connectivity aberrancies. Even though there was convergence towards a single signature, several connectivity subtypes were identified. Our results define four prominent network dysfunction modes of cross-etiological relevance for ASD, and highlight a pivotal contribution of etiological variation to connectivity heterogeneity in autism as hinted by previous human studies [72, 73].

## Materials and methods

### Ethical statement

All study procedures were approved by the institutional review board at the involved medical centers and are in accordance with the ethical standards of the Declaration of Helsinki of 1975, as revised in 2008. All experiments performed at ETH Zürich were in accordance with the Swiss federal guidelines for the use of animals in research, and under licensing from the Zürich Cantonal veterinary office. All experiments performed at IIT Rovereto were conducted in accordance with the Italian Law (DL 27/1992 and DL 26/2014, EU 63/2010, Ministero della Sanità, Roma to A.Go.) and the recommendations in the Guide for the Care and Use of Laboratory Animals of the National Institutes of Health. Animal research protocols were also reviewed and consented to by the respective animal care committees.

### Magnetic resonance imaging

Data acquisition was performed in both sites on a Biospec 70/16 small animal MR system (Bruker BioSpin MRI, Ettlingen, Germany). Scans from ETH Zürich were obtained with a cryogenic quadrature surface coil (Bruker BioSpin AG, Fällanden, Switzerland). Scans from IIT Rovereto were obtained with a 72 mm birdcage transmit coil and a custom-built saddle-shaped four-element coil for signal reception. Common standard adjustments included calibration of the reference frequency power and the shim gradients using MapShim (Paravision v6.1). BOLD rsfMRI time series were acquired using an Echo Planar Imaging (EPI) sequence, harmonized between the two sites. Sequence details from different sites/models are described in the appropriate references from Table 1. In all datasets, mild anesthesia levels were maintained using either isoflurane (0.5%) +medetomidine (0.05 mg/kg) for data acquired at ETH or halothane (0.75%) for data acquired at IIT and were based on published protocols optimized for maintaining physiological stability, and include intubation and mechanical ventilation [19, 27–29].

### Data preprocessing and connectome construction

Resting-state fMRI datasets were commonly preprocessed using a standard minimal-preprocessing pipeline [30]. Briefly, the nuisance model used for signal regression includes six head motion parameters and ventricle signals. No global signal regression was used, as this pre-processing step introduces spurious anti-correlations and strongly blunts long-range functional connectivity in rodents [30]. Thereafter, datasets were de-spiked, band-pass filtered, skull-stripped and normalized first to an EPI study-specific template and then to the Allen Brain Institute reference atlas (http://mouse.brain-map.org/static/atlas) using ANTs v2.1 (http://picsl.upenn.edu/software/ants/). BOLD time series were extracted using the Allen Reference Atlas ontology and their connectivity couplings were measured using *Z* scored regularized Pearson’s correlation coefficient (FSLNets). 165 ROIs in both hemispheres were included in the rsfMRI analysis. This included regions in isocortex, olfactory areas, hippocampal formation, cortical subplate, striatum, pallidum, thalamus, hypothalamus, midbrain and pons (Fig. 1A).

### Cluster definition and verification

All statistical analyses and clustering were performed using Matlab (R2019a) and Python (Scikit-learn, UMAP) [74, 75]. We first sought to reduce the complexity of the full connectome (165 × 165 ROIs resulting in 13530 edges) by considering only the strongest, positively-correlated edges. We therefore applied a sparsity threshold to the connectivity matrix. This strategy therefore does not take into account anticorrelated regions. Based on an analysis in non-transgenic mice (see “Results”), we choose a sparsity of 4% because that ensures high similarity across the different cohorts. This level of sparsity led to the inclusion of 545 connections (i.e., edges). For each of these edges and in each of the 16 ASD mouse models, we quantified deviations from the wildtype’s littermate mean connectivity strength in form of Cohen’s d effect size estimates.

The effect size vectors were then fed into a Gaussian Mixture Model (GMM) clustering algorithm to establish the links between mouse cohorts based on their effect sizes. GMM treats the data as a superimposition of multiple Gaussian distributions and it applies the Expectation-Maximization (EM) algorithm to determine the mean and the variances of these distributions (the latter distinguishes GMM from other clustering algorithms, such as k-means).

To determine the consistency of the clustering, we used a bootstrapping procedure to generate a distribution of the real data which we obtained by applying the GMM after randomly removing 20% of the data in each cohort. We compared this against a null model, which was obtained by randomly shuffling the labels of mutant versus WT mice of each cohort. This operation was repeated 1000 times to determine the consistency of the clustering (1000 times). For each iteration, we calculated the effect-size in every edge and applied GMM clustering as described above. The proportion of time (using 1000 repeats) that two mouse cohorts were grouped together was compared between the bootstrapped and null distributions and used to determine similarity across cohorts in the clusters.

To establish the best cluster solution, we computed the silhouette value. The silhouette value ranges from −1 to 1. A high silhouette value indicates that models are well matched to their own cluster, and poorly matched to other clusters. We measured silhouette scores for different numbers of clusters (repeated 1000 times) and compared the values between bootstrapped and null distributions.

To assess connectivity profiles on a mid-level scale, the number of edges that turned significant (randomized, nonparametric statistics, p < 0.05) in/out of a given anatomic structure were summed (node-level comparison). Finally, for the coarsest-scale analysis, anatomic regions were collapsed (summed) into their parent structures according to the Allen Mouse Brain Common Coordinate Framework (CCFv3) ontology (parent-level comparison).

For statistical analysis of connectome edge-strength distributions, we used a non-parametric permutation test using FSL randomize (*n* = 5000 repeats) corrected for multiple comparisons using false detection rate (FDR).

## Supplementary information

Legends to supplementary Figures

Supplementary_Figure 1

Supplementary_Figure 2

Supplementary_Figure 3

Supplementary_Figure 4

Supplementary_Figure 5

Supplementary_Figure 6

Supplementary_Figure 7

Supplementary_Figure 8

Supplementary_Figure 9

## Data Availability

Raw data and codes used to generate the findings of this study are available from the first author VZ, corresponding author AG, and NW on request.
