# Correction: Brain mapping across 16 autism mouse models reveals a spectrum of functional connectivity subtypes

**DOI:** 10.1038/s41380-022-01510-0

**Published:** 2022-03-23

**Authors:** V. Zerbi, M. Pagani, M. Markicevic, M. Matteoli, D. Pozzi, M. Fagiolini, Y. Bozzi, A. Galbusera, M. L. Scattoni, G. Provenzano, A. Banerjee, F. Helmchen, M. A. Basson, J. Ellegood, J. P. Lerch, M. Rudin, A. Gozzi, N. Wenderoth

**Affiliations:** 1 Neural Control of Movement Lab, ETH Zurich, Zurich, Switzerland; 2 Functional Neuroimaging Lab, Istituto Italiano di Tecnologia, Center for Neuroscience and Cognitive Systems, Rovereto, Italy; 3 Laboratory of Pharmacology and Brain Pathology, Neurocenter, Humanitas Clinical and Research Center - IRCCS, Rozzano, Mi Italy; 4 CNR Institute of Neuroscience, Milano, Italy; 5 Department of Biomedical Sciences, Humanitas University, Pieve Emanuele Milan, Italy; 6 F.M. Kirby Neurobiology Department, Boston Children’s Hospital, Harvard Medical School, Boston, MA USA; 7 Center for Mind/Brain Sciences (CIMeC), University of Trento, Rovereto, Italy; 8 Research Coordination and Support Service, Istituto Superiore di Sanità, Rome, Italy; 9 Department of Cellular, Computational and Integrative Biology. (CIBIO), University of Trento, Trento, Italy; 10 Brain Research Institute, University of Zurich, Zurich, Switzerland; 11 Biosciences Institute, Newcastle University, Newcastle upon Tyne, UK; 12 Centre for Craniofacial and Regenerative Biology, King’s College London, London, UK; 13 MRC Centre for Neurodevelopmental Disorders, King’s College, London, London, UK; 14 Mouse Imaging Ctr., Hosp. For Sick Children, Toronto, ON Canada; 15 Department of Medical Biophysics, University of Toronto, Toronto, ON Canada; 16 Wellcome Centre for Integrative Neuroimaging, FMRIB, Nuffield Department of Clinical Neurosciences, University of Oxford, Oxford, UK; 17 Institute for Biomedical Engineering, University and ETH Zurich, Zurich, Switzerland

**Keywords:** Autism spectrum disorders, Neuroscience

Correction to: *Mol Psychiatry* 10.1038/s41380-021-01245-4, Published online 11 August 2021

Table 1 lacks important phenotypic information regarding the Syn2 mouse line (Syn2), and also reports a set of wrong references. We apologies for this oversight, which was due to misalignment of text and table referencing software.

**Table 1:** Available datasets.

ASD mouse model	Control strain	Sex	Age (weeks)	Behavioral traits*	Construct validity^†^	References	Scan site
16p11.2 (df/+)	129/C57BL6J	Males	18–20	3, 5	c	11	IIT
BTBR T+tf/J	C57BL/6J	Males	26	1, 2, 3, 4, 5	d	21	IIT
CDKL5 KO	C57BL/6J	Males	23–25	3, 5	b, f	–	ETH
CDKL5 Het (+/−)	C57BL/6J	Females	17–19	3, 5	b, f	–	ETH
CHD8	C57BL/6J	Mixed	15–18	3, 4, 5	a	26	IIT
CNTNAP2_ETH (−/−)	C57BL/6J	Mixed	14–16	1, 4, 5	a, f	16	ETH
CNTNAP2_IIT (−/−)	C57BL/6J	Males	13–14	1, 4, 5	a, f	23	IIT
En2 (−/−)	C57BL/6J	Mixed	12–16	1, 4, 5	a, e, d	24	ETH
FMR1.1 (−/y)	C57BL/6J	Males	14–16	1, 2, 3, 4	b, f	16	ETH
FMR1.2 (−/y)	C57BL/6J	Males	13–15	1, 2, 3, 4	b, f	22	ETH
IL6	C57BL/6J	Mixed	14	1, 4	d	76	ETH
Mecp2 Het (+/−)	C57BL/6J	Females	12–14	1, 4, 5	b, f	–	ETH
SGSH (−/−)	C57BL/6N	Mixed	20–22	5	b	–	ETH
SHANK3b ex4-9	C57BL/6J	Males	19–21	1, 2, 3, 4, 5	a, f	25	IIT
Syn2	C57BL/6J	Males	25–31	1, 2, 3, 4	f	56	IIT
TREM2 (−/−)	C57BL/6J	Mixed	12–15	3, 4	d	37	ETH

*Behavioral traits related to ASD reported by others in selected models: (1) social behavior deficits; (2) reduced vocalizations; (3) repetitive behavior; (4) cognitive behavior; (5) motor performance.

^†^Construct validity includes: (a) Genetic association; (b) Related syndrome; (c) Autism copy number variants; (d) Autistic-like behavior; (e) Serotonin-related phenotype; (f) Synapse related.

The original article has been corrected.

