# Supplementary material for: Brain mapping across 16 autism mouse models reveals a spectrum of functional connectivity subtypes
Source: Mol Psychiatry. 2021 Aug 11;26(12):7610–20. doi: 10.1038/s41380-021-01245-4 (PMC8873017; doi:10.1038/s41380-021-01245-4)
Supplement: Supplementary file 1 — Legends to supplementary Figures [file 41380_2021_1245_MOESM1_ESM.docx]

**Brain mapping across 16 autism mouse models reveals a spectrum of functional connectivity subtypes**

**Legend to supplementary Figures**

***Supplementary Figure 1****. A Support Linear Vector Machine approach was used to assess whether a classifier trained with half of the wildtype data (train-set) could distinguish the site of origin in the other half of the data (test-set). We therefore: (i) Computed the functional connectome in all wildtype animals separately. (ii) Used a supervised ML approach and trained a linear support vector machine (LSVM) on half of the data to distinguish the site of origin of individual datasets. (iii) Tested the accuracy of the classifier in the other half of the data (test-set) and measured the balanced classification accuracy. (iv) Compared the balanced accuracy of the test dataset against a null model generated by shuffling the site-labels. The train-test process was repeated 100 times at multiple sparsity levels (2% to 20%). The LSVM failed to predict the site of origin of the test dataset for all sparsity levels. Please note that we varied the parameters of LSVM such as the regularization parameter ('C': [0.01,0.1,1, 10, 100, 1000]) or the Kernel coefficient ('gamma': [10,1,0.1,0.01,0.001,0.0001]) across a broad range of values. In all cases, the algorithm could not distinguish between the sites in the test dataset above chance level (not shown).*

***Supplementary Figure 2****. Averaged Euclidian distance within- and between-groups in the UMAP two-dimensional, three-dimensional and ten-dimensional embeddings. A) In transgenic animals, we found a reduced distance between animals of the same cohort compared to animals of other cohorts, suggesting that similar connectivity deviations depend on the etiology of the model. B) However, this did not occur in wildtype animals, suggesting that they have a similar functional connectome organization which is not influenced by the site of origin, and therefore on the anesthesia protocol used for this study.*

***Supplementary Figure 3.*** *UMAP 2-dimensional embedding of the connectome data from the 176 individual transgenic and 174 wildtype animals of the AMC dataset. Individual data are Z-scored and normalized to the average cohort’s wildtype control population. The shape of the elements represents the cohort, while the colors represent the clusters.*

***Supplementary Figure 4.*** *A-F) Clustering probability matrices obtained after varying the sparsity thresholding of the connectome template. For each sparsity level, we calculated the probabilities for each pair of models to be clustered together using a gaussian mixture model repeated 1000 times. The clustering probabilities were compared against an appropriate null model of data with the same sparsity level but with randomly shuffled labels. G) Pearson’s Rho ranged between 0.962 and 0.878, indicating that clustering probabilities are not strongly influenced by sparsity levels.*

***Supplementary Figure 5.*** *A-C) Clustering probability matrices obtained using a set of 165 equally sized ROIs using spheres with a diameter of 3 voxels. The ROIs are centered on the center of gravity of each region of the Allen Brain atlas. D) The clustering probabilities using the original Allen Brain Parcellation and the new equally sized ROIs show strong similarities (Pearson’s r = 0.9620, p<0.00001).*

***Supplementary Figure 6.*** *A-C) Clustering probability matrices obtained using a new parcellation scheme of 165 ROIs taken separately in each hemisphere (330 ROIs in total). A) The connectome analysis in wild type mice confirmed that the similarity between the data sets is maximal for a sparsity threshold of 4%. B-E) The probabilities of clustering using this bilateral parcellation scheme are similar to those reported in Figure 3 (Pearson's rho = 0.9231), suggesting that the use of edges that include information on interhemispheric connectivity does not change the nature and the interpretation of our results. F) Analysis of clustering similarities in single cohorts found significant differences in clustering similarities only in the BTBR mouse model. Notably, BTBR mice are less likely to be grouped with any other mouse model. We believe this is due to the fact that interhemispheric connectivity is severely impaired in acallosal BTBR mice.*

***Supplementary Figure 7:*** *edge-edge and parent-level statistics show a brain-wide heterogeneous distribution of under- and over-connectivity across the four clusters.*

***Supplementary Figure 8****. Edge-edge and parent-level statistics indicate under- and over-connectivity deficits that are unique to one cluster as compared to the other three. This analysis highlights the unique profile of each cluster. The ASD-etiologies in Cluster 1 are characterized by prominent over-connectivity in the prefrontal cortex, posterior parietal, olfactory area, cortical subplate, striatum and pallidum. This was in complete opposition to the alterations observed in Cluster 4, which are characterized instead by under-connectivity in the same areas, but also by a strong over-connectivity within the somatomotor areas and between midbrain and thalamus. Cluster 2 and 3 instead showed increased connectivity in hippocampus and decreased connectivity in pons, or a marked reduction in connectivity in pons and hippocampus, respectively.*

***Supplementary Figure 9.*** *Non-parametric statistics revealed a handful of connectivity deficits that are common across all models. Circos plot illustrates that the majority of the significant edges have under-connectivity between insula, somatosensory cortex, stratum, amygdala. Blue colors represent under-connectivity. Red colors represent over-connectivity as compared to wildtype littermates. AIp = Anterior Insula, posterior, Cla=claustrum, CPu = caudoputamen, DG = dentate gyrus, EPd= Endopiriform nucleus, dorsal part, GU=gustatory area, MOp=primary motor area, PAR= parasubiculum, SCm= superior colliculus, motor related, SSp-ul= primary somatosensory area, upper limb, SSs = supplementary somatosensory area.*
