# Supplementary figures and images for: Brain mapping across 16 autism mouse models reveals a spectrum of functional connectivity subtypes
Source: Mol Psychiatry. 2021 Aug 11;26(12):7610–20. doi: 10.1038/s41380-021-01245-4 (PMC8873017; doi:10.1038/s41380-021-01245-4)

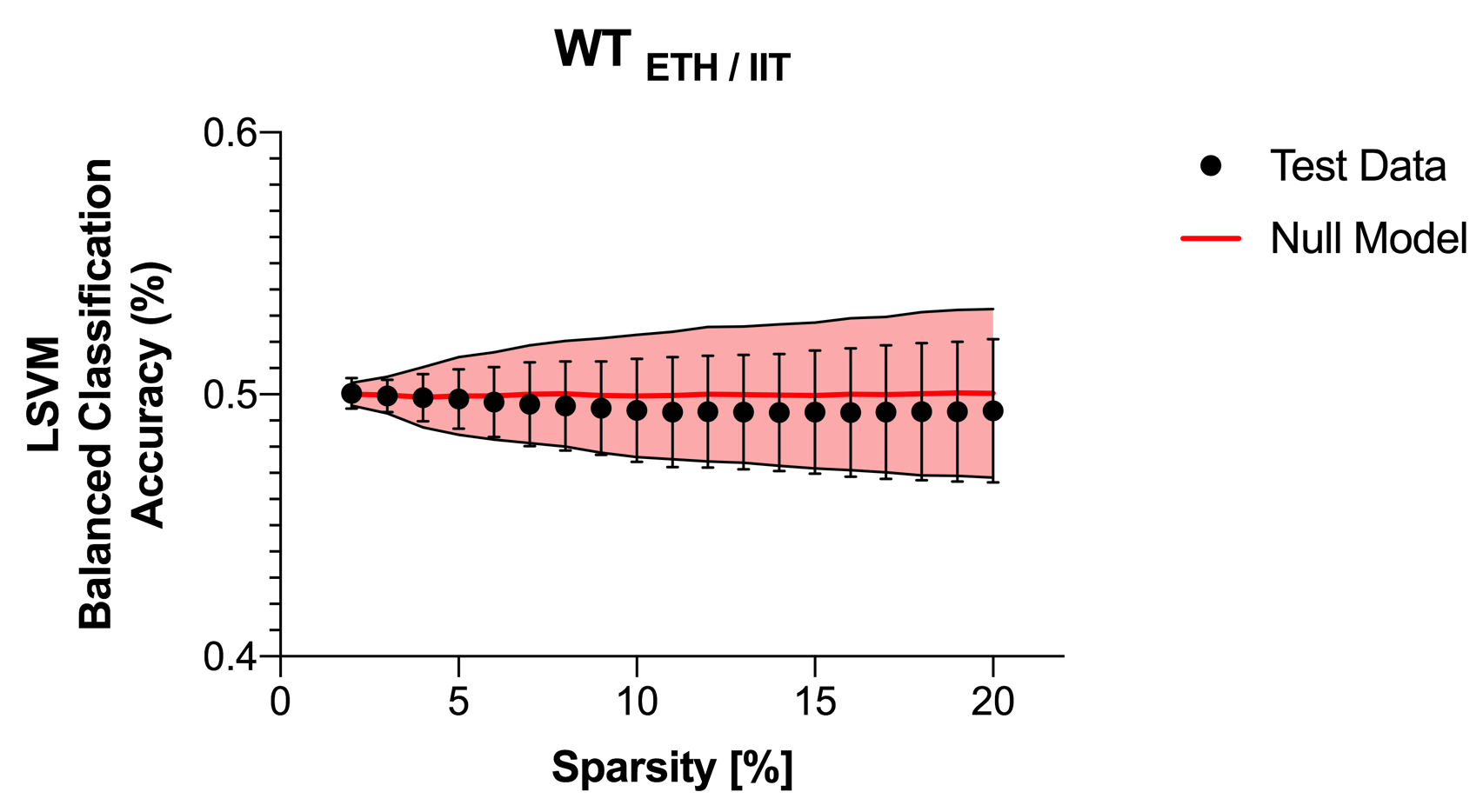

Supplement: Supplementary file 2 — Supplementary_Figure 1 [file 41380_2021_1245_MOESM2_ESM.png]

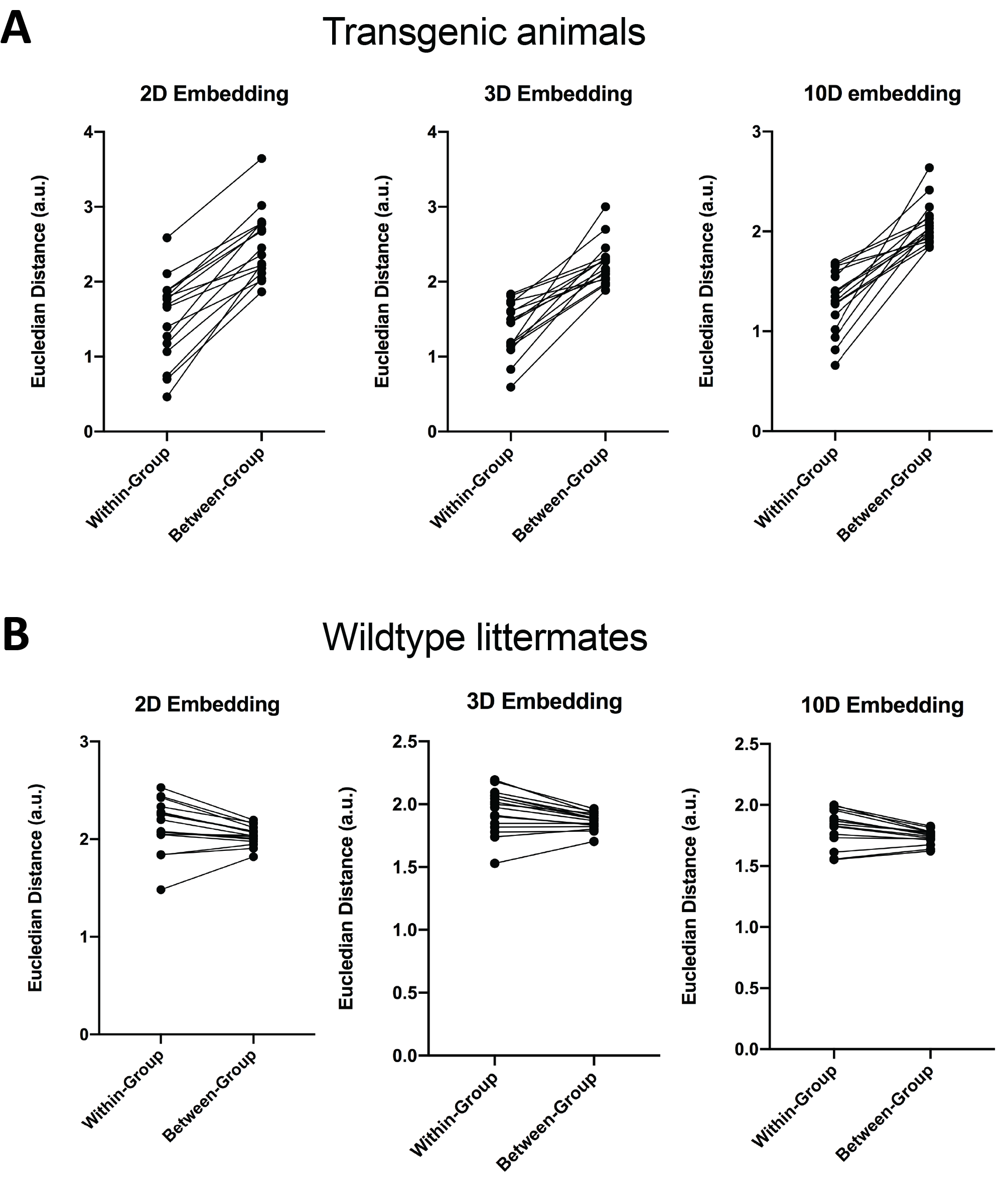

Supplement: Supplementary file 3 — Supplementary_Figure 2 [file 41380_2021_1245_MOESM3_ESM.png]

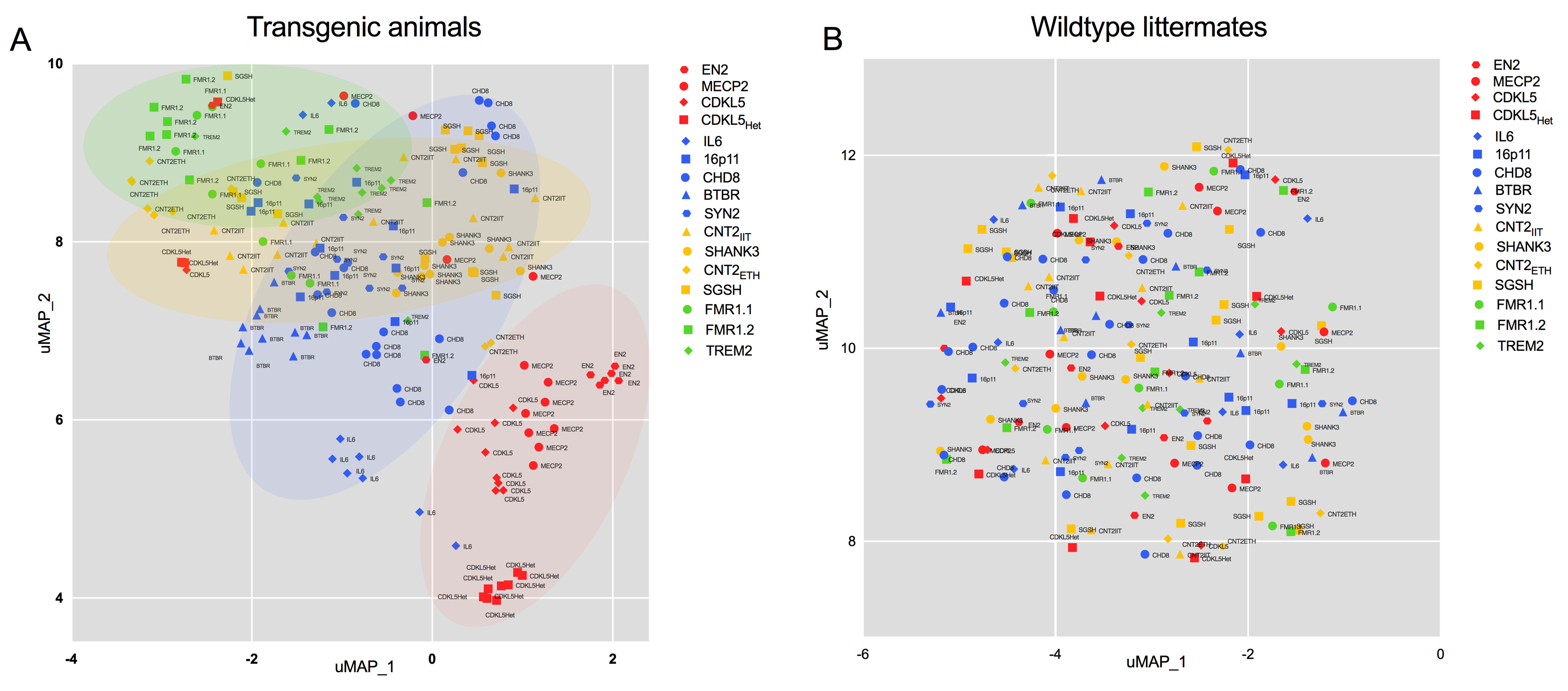

Supplement: Supplementary file 4 — Supplementary_Figure 3 [file 41380_2021_1245_MOESM4_ESM.png]

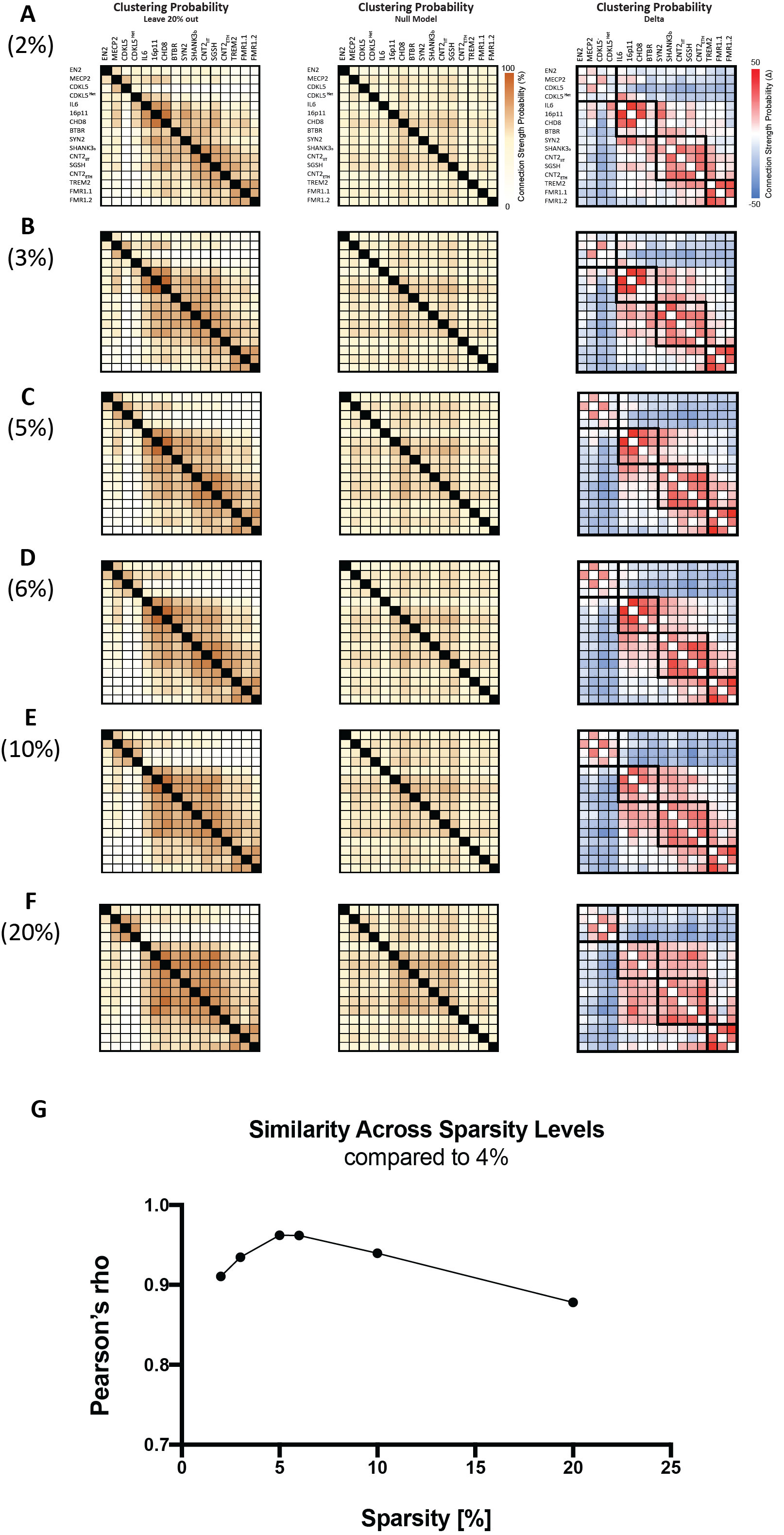

Supplement: Supplementary file 5 — Supplementary_Figure 4 [file 41380_2021_1245_MOESM5_ESM.png]

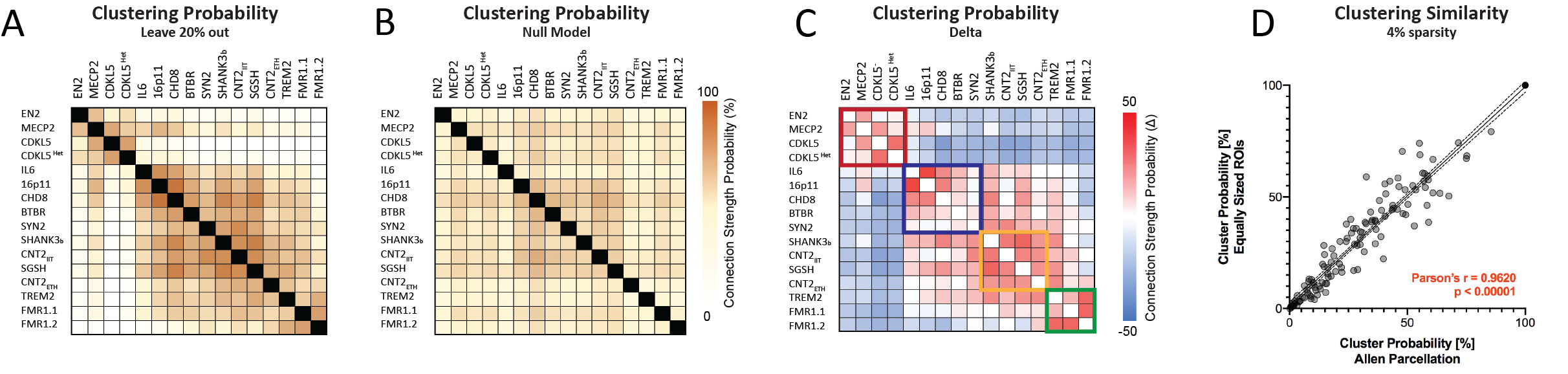

Supplement: Supplementary file 6 — Supplementary_Figure 5 [file 41380_2021_1245_MOESM6_ESM.png]

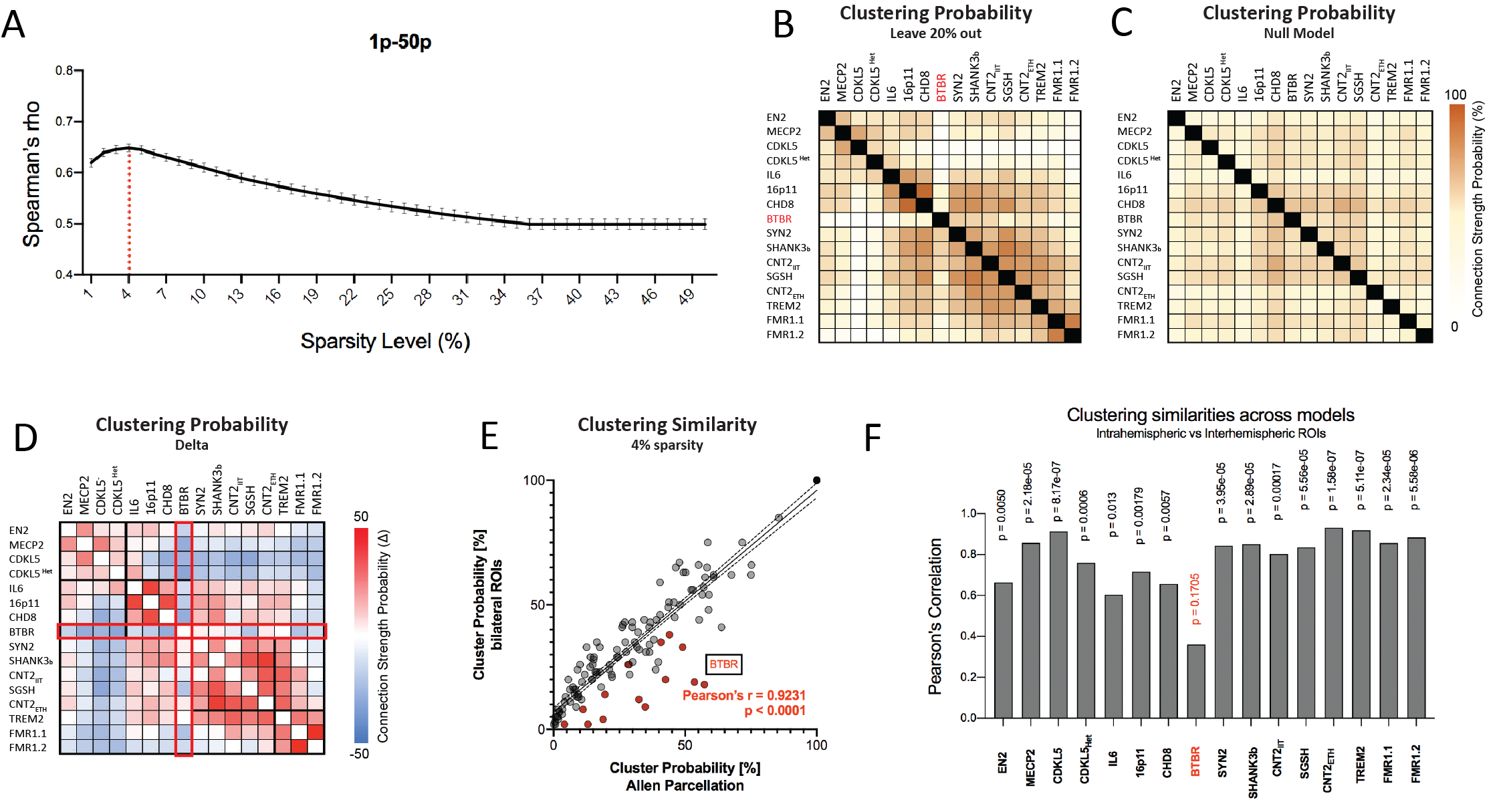

Supplement: Supplementary file 7 — Supplementary_Figure 6 [file 41380_2021_1245_MOESM7_ESM.png]

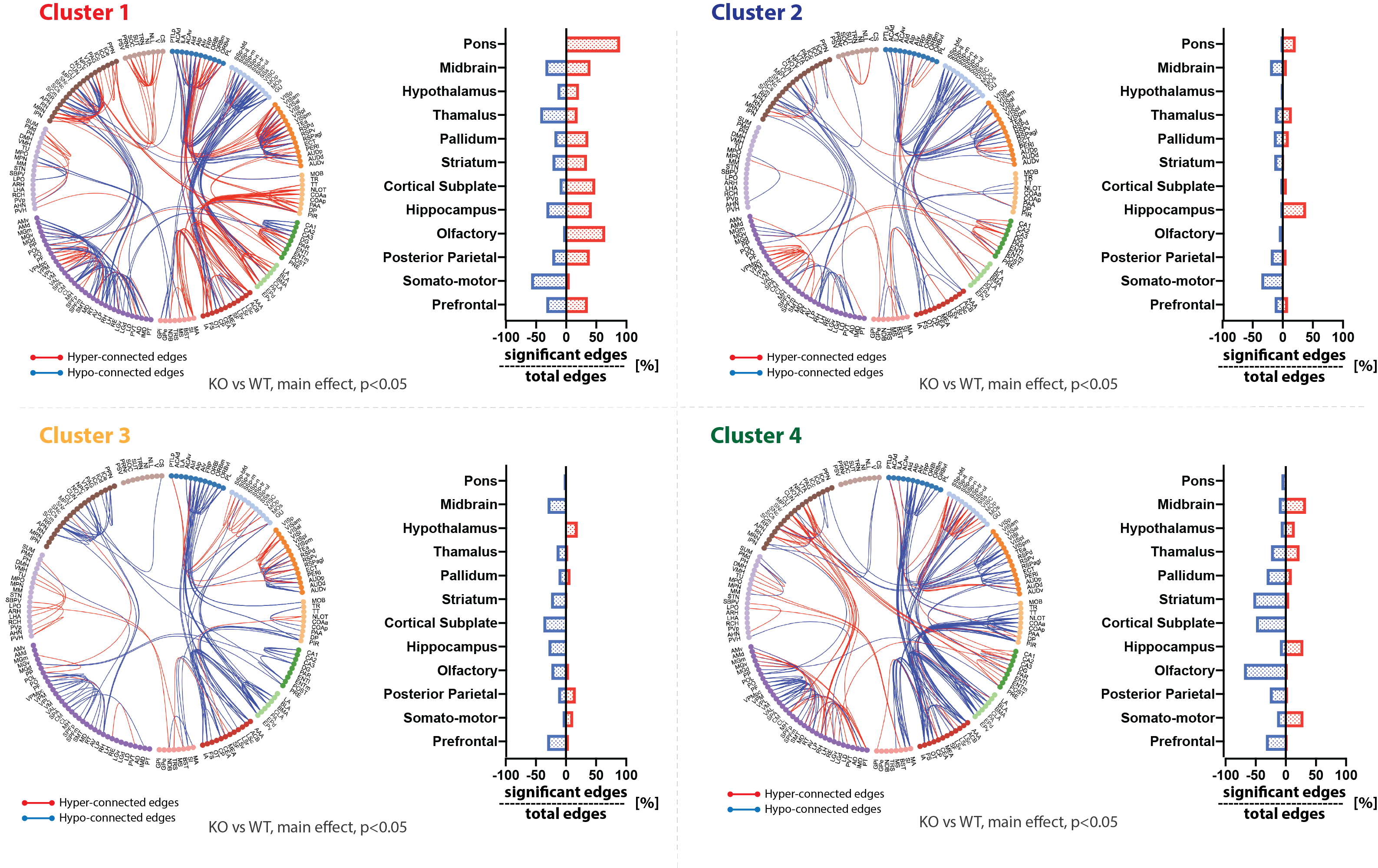

Supplement: Supplementary file 8 — Supplementary_Figure 7 [file 41380_2021_1245_MOESM8_ESM.png]

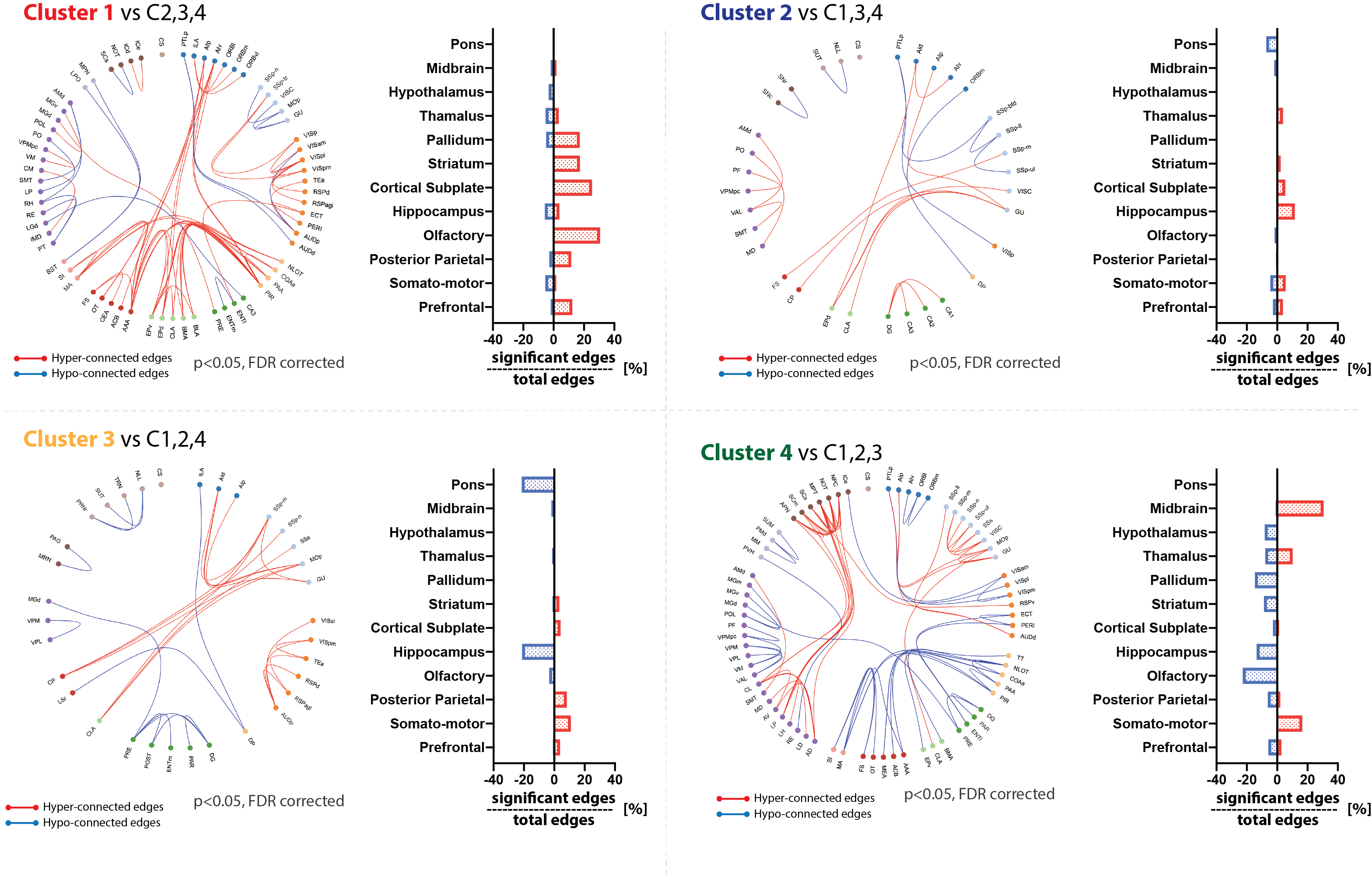

Supplement: Supplementary file 9 — Supplementary_Figure 8 [file 41380_2021_1245_MOESM9_ESM.png]

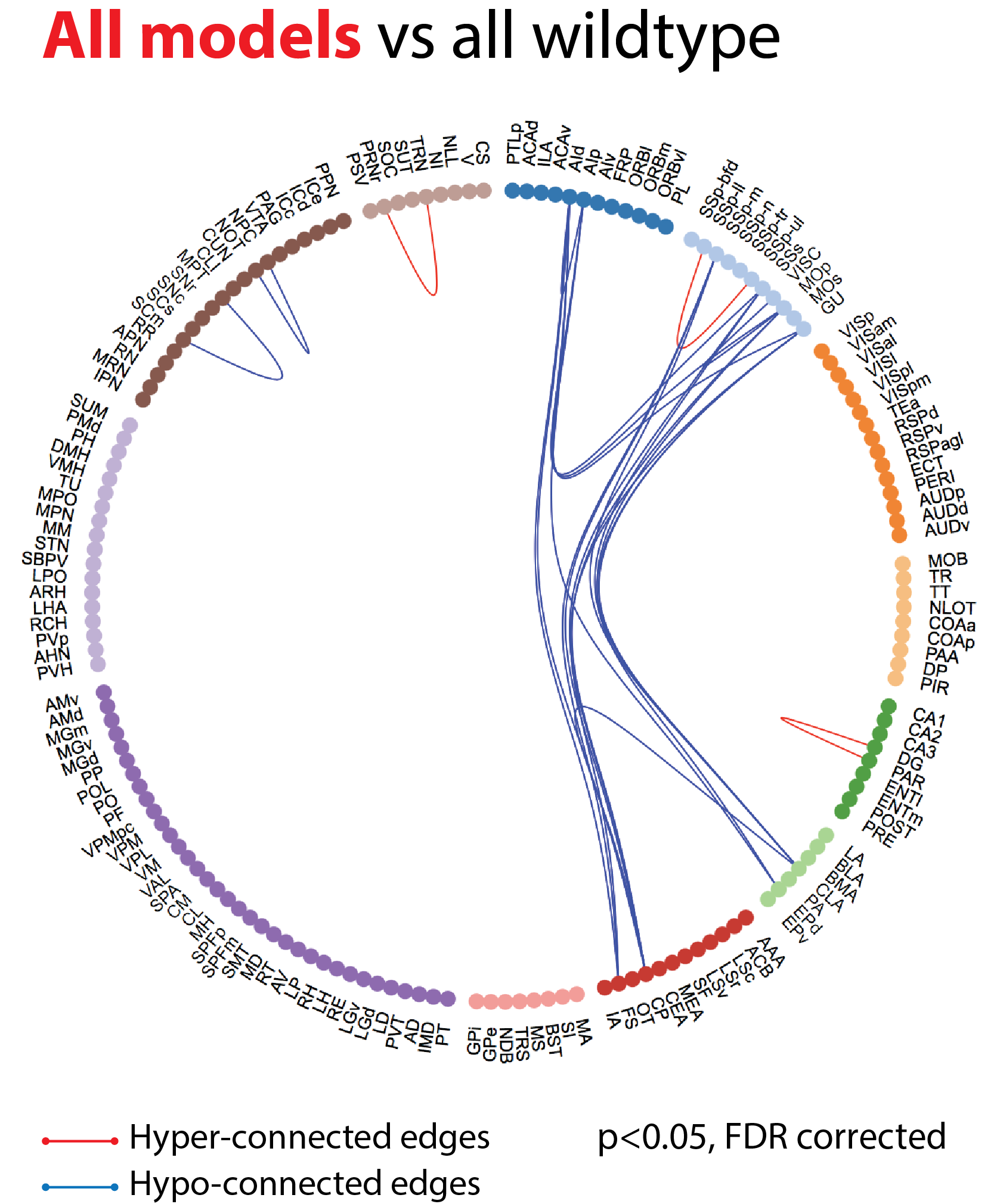

Supplement: Supplementary file 10 — Supplementary_Figure 9 [file 41380_2021_1245_MOESM10_ESM.png]
